# The Bayesian boom: good thing or bad?

**DOI:** 10.3389/fpsyg.2014.00765

**Published:** 2014-08-08

**Authors:** Ulrike Hahn

**Affiliations:** Department of Psychological Sciences, Centre for Cognition, Computation, and Modelling, Birkbeck, University of LondonLondon, UK

**Keywords:** Bayesian modeling, rationality, normativity, probability

## Abstract

A series of high-profile critiques of Bayesian models of cognition have recently sparked controversy. These critiques question the contribution of rational, normative considerations in the study of cognition. The present article takes central claims from these critiques and evaluates them in light of specific models. Closer consideration of actual examples of Bayesian treatments of different cognitive phenomena allows one to defuse these critiques showing that they cannot be sustained across the diversity of applications of the Bayesian framework for cognitive modeling. More generally, there is nothing in the Bayesian framework that would inherently give rise to the deficits that these critiques perceive, suggesting they have been framed at the wrong level of generality. At the same time, the examples are used to demonstrate the different ways in which consideration of rationality uniquely benefits both theory and practice in the study of cognition.

## Introduction

The last two decades of cognitive science have seen a bit of a revolution: probabilistic models of cognition, in particular, Bayesian models have not only steadily increased in volume, but have come to grab a large market share in those outlets, such as Psychological Review, that focus on psychological “theory.” These trends are manifest not just in a wealth of reviews (e.g., Chater et al., 2006, 2010) and bibliometric statistics, but, last but not least, in the fact that Bayesian models have recently prompted a number of high-profile critiques (e.g., Elqayam and Evans, 2011; Jones and Love, 2011; Bowers and Davis, 2012a,b). A pre-requisite to critique is getting noticed in the first place, and, given that these critiques concern formal, mathematical models of cognition, that is no mean feat.

So these critiques may plausibly be taken to signal a moment of arrival in the development of the paradigm, particularly given that they were written for a general audience, not just for specialists within the discipline. At the same time, it seems likely that these critiques provide insight that research would be well-advised to heed. In light of this, the present paper scrutinizes these recent critiques with a view to identifying the key implications they present for future work.

## Fundamental critiques

Three sets of criticisms have recently been aimed at Bayesian models of cognition: the target article in Behavioral and Brain Sciences by Jones and Love (2011) raising the specter of “Bayesian fundamentalism,” Bowers and Davis article in Psychological Bulletin (2012) on “Bayesian just-so stories” and, from an even broader perspective, Elqayam and Evans (2011) recommendation to abandon a central role for normative models in the study of the cognition. While there is some overlap between these critiques, each makes distinct points. Each is also a lengthy article in its own right, containing a wealth of observations and claims. However, for the purposes of this article, four main claims of interest will be highlighted and addressed for each.

### Jones and Love (2011)

Jones and Love find that rational Bayesian models are (1) significantly unconstrained, because they are generally uninformed by either process-level data or environmental measurement. Furthermore, (2) the psychological implications of most Bayesian models are also unclear (last but not least because there is little contact with mechanism or process). The retreat to the level of abstraction away from process at which Bayesian models are typically phrased is not perceived to be of intrinsic interest because (3) Bayesian inference itself is conceptually trivial (Bayes' theorem is just a simple “vote counting”). And finally, (4) many Bayesian models simply recapitulate existing (mechanistic level) theories.

### Bowers and Davis (2012a,b)

Here it is maintained that (1) flexibility with priors, likelihoods, and utility functions frequently makes models unfalsifiable, while (2) Bayesian theories are also rarely better at predicting data than alternative (and simpler) non-Bayesian ones. In general, for understanding cognition and building insightful models of cognitive processes, (3) constraints other than rational analysis are more important. As a consequence, (4) psychology and neuroscience now abound with Bayesian “just so” stories, that is, mathematical analyses of cognition that can be used to explain almost any behavior as optimal.

### Elqayam and Evans (2011)

The focus of Elqayam and Evan's critique, finally, is more general in its target than just Bayesian modeling, affecting also the use of decision-theory and logic as other putative norms of rationality. The central point in Elqayam and Evan's paper is (1) a critique of what they call “normativism”: the idea that human thinking reflects a normative system against which it should be measured and judged. Normativism is conceptually dubious because it invites fallacious is-to-ought and ought-to-is inferences (2). At the same time, little can be gained from normativism that cannot be achieved by descriptivist computational-level analysis (3). As a consequence, Elqayam and Evans believe that (4) theories of higher mental processing would be better off if freed from normative considerations.

Each of these articles has already seen extensive counter-critique, last but not least the open peer commentaries that are an integral part of the journal format for two of these three articles (and for the third, Bowers and Davis, 2012a,b), see the reply in the same journal by Griffiths et al., 2012). It is the contention of the present paper, however, that there are still things to be said on this topic, and that some things that have been said deserve to be said again and become clearer or more compelling when put together in a single overall argument. First and foremost, it is the contention of this paper that closer consideration of actual examples of Bayesian treatments of different cognitive phenomena allows one to defuse the above critiques. Specifically, it will be argued that one of the main reasons the critiques go amiss is that they have been phrased at the wrong level of generality. More detailed consideration of specific examples, however, is not something the restrictive format of open peer commentary readily supports.

## The diversity of bayesian modeling

One of the tensions in all three critiques is that, while it is likely they have been motivated by particular applications, they are pitched as general critiques of a paradigm. This is striking because Bayesian probability itself is, in first instance, a formalism, that is, a “language.” As such, it affords many and diverse applications. How then could such a diverse set of applications suffer from common problems? For one, it could do so coincidentally: researchers who avail themselves of this language happen to, by and large, be researchers who are comparatively poor at the task of model-building. For example, they may fail to appreciate fundamental criteria of “goodness” for a model that a field has managed to identify. The root cause, in this case, is effectively sociological. There is nothing within the formalism itself that makes necessary the deficits observed, and, in the hands of others, these limitations could easily be rectified. The second possibility is that there is some deeper limiting factor in the formalism that is responsible for the perceived limitations. In this latter case, the formalism itself is indeed, at least partly, to blame. Both cases would merit critique, but the nature of that critique, in order to be appropriate and hence constructive, would have to be very different. The only way to distinguish between these two possibilities is to consider specific examples. Limitations of the formalism itself should emerge as common aspects of all examples considered.

**Figure 1:**
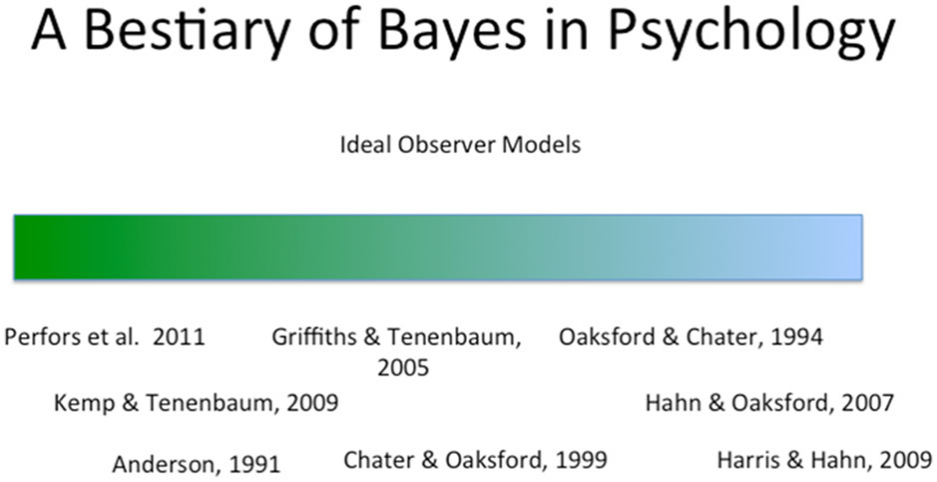
**The figure presents examples of applications of Bayesian modeling**. The examples, discussed in the text, reflect both different types of application in terms of the aspect of the cognitive system modeled, and the theoretical and methodological role accorded to Bayesian inference as a result.

For these purposes it is important to consider a broad range of examples. Figure 1 contains a set of such examples, chosen with diversity in mind. The list contains both some of the most famous and influential Bayesian modeling (e.g., Anderson, 1991; Oaksford and Chater, 1994) and other examples, which, by comparison, are completely obscure (e.g., Harris and Hahn, 2009). The examples vary also in the cognitive domain to which the model is applied, ranging from judgment through reasoning and argumentation to categorization and language acquisition.

In fact, these differences in domain give rise to an informal ordering within the Figure: the green-blue dimension^1^. This dimension may, in first instance, be taken to reflect the extent to which the underlying cognitive task *inherently involves inference, and more specifically, probabilistic inference*.

To illustrate: On the far right hand end of the “blue spectrum,” the task participants face in Harris and Hahn's (2009) studies of evidential coherence is that of evaluating, from the perspective of the police, the potential location of a body given the testimony of (less than perfectly reliable) multiple witnesses. Not only is this inherently an inferential task involving uncertainty, but participants are specifically asked to evaluate a question about ‘how likely it is’ that the body lies within a particular area on a map.

By contrast, at the green end, Anderson's (1991) famous rational model of unsupervised categorization addresses the task of imposing categories on unlabeled instances, that is, partitioning a set of objects into distinct classes of object. This need not be viewed as an inference task at all. Furthermore, even if the task is to be construed as one involving inference, there is a wealth of different choices concerning what that inference may be about. Ultimate answers to the fundamental question of what unsupervised categorization does and what it is for rest on extremely difficult questions about the relationship between mind and world (e.g., the extent to which we “discover” categories in the world or instead impose them) and the role of categories in language and thought.

In fact, rival accounts of unsupervised categorization which assume that classification proceeds on the basis of inter-item similarity, for example, may assume that such similarities reflect deep facts about the environment (or, human perceptions thereof, given that “similarity” is a subjective, not an objective relation between objects, see e.g., Hahn and Chater, 1997), or they may simply take as their point of departure that human categorization seems sensitive to similarity.

Anderson's (1991) model is based on the idea that categorization reflects the goal of optimally predicting the unseen features of objects, that is, we wish to be able to predict *P*_i_(*j|F_n_*), the probability that (as yet unseen) dimension *i* of the object possesses the value *j*, given the feature structure *F*_n_ observed so far. Categories are formed to assist this goal. Hence, objects are assigned to categories in such a way as to make the feature structures of those objects most probable. As a Bayesian model, the rational model assigns a new object to the most probable category *k* given the features observed, *P(k|F)*. In so doing, the model may choose to create an entirely new category for that item.

The fact that the two examples, Harris and Hahn's study of coherence, and Anderson's rational model, fall on opposite ends of the spectrum with regard to the extent to which the task under investigation is *necessarily* construed as involving probabilistic inference has immediate implications for the role of rational, Bayesian inference in each case.

Where the task is uncontroversially construed as an inferential one, the mapping between task and formalism is more or less direct. Where it is not, the probabilistic construal is merely one of many possible, equally plausible, task decompositions. This has direct consequences for the “normative” or “rational” status bestowed by Bayesian inference. While it is the case that Bayesian probabilistic inference has a privileged status that makes its use “rational” or “optimal” in certain well-defined senses (more on this in a moment), this normativity or rationality only goes as far as the inference itself. If the task may plausibly be construed as not involving inference in the first place, then the resultant model as a whole is neither inherently more “normative” or “rational” than any other.

Associated with the difference in role for Bayesian inference at the two ends of the green-blue spectrum are other differences. In Harris and Hahn's (2009) study prior probabilities are objectively defined within the task. There is nothing to “choose” here by the modeler, and there are no free parameters. In the case of Anderson's rational model, by contrast, model behavior is critically dependent on prior probabilities for category membership. Anderson (1991) specifies this prior in the following way:

(1) $$p(k)=\frac{cn_{k}}{(1-c)+cn}$$

where *n*_k_ is the number of objects assigned to category *k* thus far, *n* is the total number of classified objects and *c* is the so-called “coupling parameter.” This parameter governs the probability that a new instance will receive an entirely new label, *P*(0):

(2) $$p(0)=\frac{1-c}{(1-c)+cn}$$

In other words, the coupling parameter determines how readily new categories will be formed: for high values of the coupling parameter, larger clusters are favored by the prior, whereas for low values the model will favor greater numbers of smaller categories. Model behavior thus varies dramatically as a function of *c*.

Furthermore, the combinatorial explosion concerning the number of possible partitions of even fairly small sets of to-be-classified objects means that Anderson's model must rely on approximation to the optimal Bayesian estimates. Alternative approximation algorithms to Anderson's are possible (e.g., Gibb's sampling, see Geman and Geman, 1984) or particle filters (see e.g., Doucet et al., 2001), and, as Sanborn et al. (2010) demonstrate, will give rise to differences in model predictions.

This makes it fuzzier what the rational model actually *is*, and makes the model harder to test empirically. However, contrary to concerns about Bayesian models articulated by Bowers and Davis (2012a,b) there is no sense in which the rational model is unfalsifiable. One can readily evaluate model predictions across values of the coupling parameter and contrast those predictions with human behavior (as in Sanborn et al., 2010) and in that way compare the rational model with competing formal models of unsupervised categorization (as in Pothos et al., 2011), and one can do this for different approximation algorithms.

Needless to say, in the case that other models perform better on such tests (as Pothos et al., indeed find them to do), no one would take that to indicate that participants' views on classification are “irrational.” Because there are so many ways the goals of categorization can be construed, the model does not prescribe what people *should* do in any strong sense. Deviating from it is not an “error” in the same way that prominent inferential failures in the judgment and decision-making literature (such as the conjunction fallacy, Tversky and Kahneman, 1983) are viewed as errors—an issue we return to below.

Concerning the critical challenge surrounding model falsifiability it seems important to distinguish vague predictions from model flexibility. Vagueness means that it is unclear exactly what predictions are, and what empirical evidence might or might not meet them. Flexibility, by contrast, means that a model or theory can change its predictions depending on parameterization; given a particular set of parameters, however, predictions are specific. The rational model not only has an important free parameter, but due to the nature of its approximation algorithm, also has stochastic variation in its model output; however, by averaging over model runs, specific predictions can be derived, and—as has been demonstrated empirically (see e.g., Pothos et al., 2011)—the model can readily be compared both with human data and with other models.

Beyond pointing out that even a flexible model such as Anderson's rational model admits falsification it is hard to know how to address Bowers and Davis claims that Bayesian models may frequently be unfalsifiable given their flexibility with priors, liklihoods and utility functions. It seems hard to see that Bayesian models are more flexible than other mathematical models that admit of parameterization. They are certainly not inherently more flexible, because in many contexts (certainly toward the “blue end” of Figure 1), Bayesian models of the task can and have been applied (and compared with human performance) without free parameters at all, because parameters such as priors or likelihoods are derived from participants estimates or because they are taken directly from environmental quantities and the model itself consists simply of Bayes theorem. In addition to the Harris and Hahn (2009) paper, other examples here include Harris et al.'s (2012) study on argumentation, and the extensive body of research within the 1960's that examined experimentally human belief revision using simple devices such as colored pokerchips drawn from bags of varying chip composition (see e.g., Peterson and Beach, 1967; Edwards, 1968). At the very least, these examples make clear that the formalism itself does not impose any particular degree of flexibility.

Other examples along the green-blue dimension fit also with the first two examples of Anderson (1991) on the one hand, and Harris and Hahn (2009) on the other. Perfors et al. (2011) simulations are aimed at addressing fundamental questions in language acquisition concerning so-called poverty of stimulus arguments, that is, arguments that seek to argue that certain aspects of language, though developmentally acquired, cannot be learned, because there is insufficient information in the linguistic input to the child (for a review and references see also e.g., Hahn and Oaksford, 2008). Perfors et al. like many researchers concerned with these questions before them (see e.g., Chomsky, 1957, 1986; Gold, 1967; Wharton, 1974) assume that the task at hand is to infer a grammar, from which the grammatical sentences of the language can be generated. However, whether this is an appropriate way to conceive of language acquisition is in itself a matter of debate. Other researchers have argued that the goal of acquisition is to learn form-meaning mappings (e.g., Bates and MacWhinney, 1989) or to learn procedures for comprehension and production (Seidenberg and MacDonald, 1999). On such views, there need be no role at all in language for a grammar as traditionally conceived. The role of Bayesian inference in Perfors et al.'s study is thus to provide an elegant, well-defined, and well-understood modeling tool. The point is not an account of what children *should* do.

Over at the “blue end” of Figure 1, however, such normative concerns are integral to Oaksford and Chater's (1994) account of Wason's selection task, a paper that, like Anderson's rational model, is a cornerstone of Bayesian modeling. Wason's classic (1968) study shows participants deviating from a falsificationist strategy when asked to select information to test a rule. While falsification was advocated as an ideal strategy for science by Popper (1959), it is not ideal in general, that is, independently of the specific hypotheses and nature of the environment as shown, for example, by Klayman and Ha (1989). And indeed, philosophers of science have not only noted that falsificationism does not capture the actual conduct of science (Kuhn, 1962; Lakatos, 1976), but have moved away from it as an ideal strategy in more recent work that adopts a Bayesian, normative perspective on scientific inference (e.g., Earman, 1992; Howson and Urbach, 1993). Oaksford and Chater (1994) seek to show that under certain simple assumptions about the structure of the environment, and certain assumptions about reasonable priors, participants' responses on the selection task are well-understood as an approximation to optimal data selection.

In general, Oaksford and Chater's treatment of conditional reasoning involves a twofold argument. On the one hand, they argue that the utility of classical logic in the context of everyday reasoning is extremely limited (see e.g., Oaksford and Chater, 1991); probability theory, by contrast, provides a natural formalism for reasoning under uncertainty. On the other hand, as they seek to demonstrate, seeming patterns of deviation in human responding on what have traditionally been conceived of as logical reasoning tasks, are well-captured under the assumption that participants view the seemingly deductive inference task as a probabilistic inference task.

This work is naturally situated toward the “blue end” as it is concerned with what are inference tasks by design. There is room for debate here on a normative level about the mapping between probability theory and the task; in particular there has been considerable philosophical debate about the appropriate formalization of the natural language condition “if … then” (see e.g., Edgington, 1995; Evans and Over, 2004), so the normative claims do not simply have to be accepted at face value. But they are integral to the overall aims of the project. At the same time, there is a descriptive component: the claim that actual participant responding is well-understood as an approximation of this normative construal. This descriptive claim may be empirically challenged, both by seeking to provide evidence of systematic deviation between model and observed behavior, and by positing alternative explanations of behavior that rest on functionally different interpretations (by participants) of the task.

Lively empirical debate has thus ensued (see e.g., the open peer commentary on Oaksford and Chater, 2009). This in itself testifies against claims about lack of falsifiability, but it is also important to note here that Oaksford and Chater's work has, in fact, brought a new level of specificity to behavioral prediction in the context of logical reasoning (see also Hahn, 2009 for discussion of this point). Prior to Oaksford and Chater's work, data in the psychology of logical reasoning were a collection of qualitative phenoma (“context effects,” “supression effects” etc.). Since their seminal (1994) paper, empirical work in the psychology of reasoning frequently involves evaluation of detailed quantitative predictions. This was first seen in Oaksford and Chater's probabilistic approach, and it is “rival approaches” that have followed in this (see e.g., Schroyens and Schaeken, 2000; Oberauer, 2006; Klauer et al., 2007).

This example speaks to a whole range of separate points in the above critiques of Bayesian models: namely, the shift to more detailed, quantitative predictions provides a ready example where Bayesian models do not simply recapitulate existing mechanism level theories [Jones and Love (4) above]; moreover, it provides an example where a Bayesian model has been “better at predicting data than simpler (non-Bayesian) alternatives” [see, Bowers and Davis, (2) above]; and it makes questionable the claim that “normativism” has hampered the development of high-level cognition so that we would be better off without it [Elqayam and Evans, (3 and 4)], and that constraints other than rational analysis are more important [Bowers and Davis (3)].

It is precisely the fact that the Bayesian framework enables quantitative prediction that enabled Oaksford and Chater's work to bring about this change in specificity of prediction within the psychology of reasoning, and their choice of formalism was driven by normative considerations. Other quantitative models may have followed subsequently, but the impulse for the shift came from the use of Bayesian modeling.

It is worth emphasis also that the reasoning tasks addressed in Oaksford and Chater's work are classic examples of “high-level cognition” which Fodor (1983) considered to be “central processing,” and hence an aspect of cognition for which we would never have detailed theories and predictions. That the field of reasoning can capture subtle changes in behavior in response to changes in the content of high-level, verbal experimental materials in such detail is thus, in and of itself, a remarkable success.

Moreover, Oaksford and Chater's treatment of selection task and logical reasoning (see also on syllogistic reasoning, Chater and Oaksford, 1999) are not alone here. Arguably, this specificity has been spreading through other aspects of human reasoning as well (see also e.g., Kemp and Tenenbaum, 2009). Hahn and Oaksford's work on informal argument fallacies are a further case in point (e.g., Hahn and Oaksford, 2007). Fallacies, or arguments that seem correct but aren't, pervade everyday informal argument. Catalogs of argumentation fallacies (also known as reasoning fallacies) originate with Aristotle and have been of concern to philosophers, logicians, and argumentation theorists to this day, though they have engendered only small amounts of psychological research in the past (e.g., Neuman and Weitzman, 2003). The longstanding goal of fallacies research has been to provide a comprehensive, formal treatment that can explain exactly why they are “bad” arguments. Hahn and Oaksford (2007) show how classic fallacies, such as the argument from ignorance (“ghosts exist, because nobody has proven that they don't”), or circular arguments (“God exists, because the Bible says so and the Bible is the word of God”) can be given a formal Bayesian treatment that distinguishes appropriately weak examples of these argument forms from ones that seem intuitively acceptable. More generally, it provides explanations of widespread intuition that arguments from ignorance or circular arguments are frequently weak: analysis across the range of possible underlying probabilities that these arguments may involve demonstrates how they are typically weaker than other types of arguments in everyday life (for details see Hahn and Oaksford, 2007).

This is in part an explicitly normative project, aimed at addressing long standing theoretical questions about the fallacies, but also more general questions about the extent to which there can be “norms” for argument quality that allow us to determine whether an argument *should* or should not convince.

At the same time, the ability to measure argument quality through use of the Bayesian, probabilistic framework allows one to generate both qualitative and quantitative predictions against which people's judgments of everyday arguments can be compared. Such comparisons have been conducted, not just in the context of the fallacies, but in the context of other arguments as well (e.g., Hahn and Oaksford, 2007; Hahn et al., 2009; Corner et al., 2011; Harris et al., 2012).

The predictions made in these contexts are not only novel, there is, in many of the cases examined, simply no alternative framework that would allow one to make predictions about the materials examined^2^. That is, the theoretical questions that can be addressed are new. But there are not just new questions about how people evaluate particular argument forms which have now been formalized. The formal framework provides a methodological tool that allows one to examine a whole host of issues concerning argumentation that are not possible without it. For example, as Corner and Hahn (2009) note, much of the communication to the public of socio-scientific issues of broad concern such as climate change, genetically modified foods, nanotechnology and so on, involves brief summaries of arguments. How people evaluate such arguments is thus a central practical concern across a broad range of issues requiring large-scale action. A normative standard for measuring argument quality, and with that participants' evaluation of arguments, provides a tool for probing whether the way people think about issues such as climate change (for example with respect to conflicting testimony, see e.g., Lewandowsky et al., 2013) differs from the way they reason in other evidential contexts. Such comparisons become possible despite the differences in argument content (and hence attendant differences in people's prior beliefs and the actual diagnosticity of the evidence) because responses to arguments from different domains can be compared *via the normative standard*: in other words, one can ask whether people's reasoning is more or less in line with normative prescriptions across different domains.

Far from re-capitulating the predictions of other, simpler, or more process-oriented models, then, this argumentation work has created a wealth of opportunity for empirical inquiry. Against the claim that other computational level theories might be as successful (or even more successful) if the limiting emphasis on normative considerations were abandoned stands the simple fact that no other computational level theory presently exists in this particular case. Given the fact that the development of the computational level theory was driven explicitly by normative considerations, it would also seem perverse to consider such considerations a block to progress [cf. Elqayam and Evans (4)], at least in this context.

Similarly, the argumentation example is at odds with the perception that “other kinds of constraints” (e.g., neural constraints) are, typically, more powerful than rational or normative considerations. And this seems indicative of “the blue end” of Figure 1 more generally. For example, it is a characteristic of Oaksford and Chater's work in the psychology of reasoning that it is precisely not concerned with process or implementation. Greater predictive power with regard to human behavior (i.e., the initial shift from qualitative to quantitative prediction) was achieved in their work despite moving to a higher level of abstraction. Moreover, the argumentation example may lead one to suspect that it is not despite that retreat to a higher level of abstraction but rather precisely because of it, that detailed quantitative predictions suddenly become possible.

What Bayesian modeling captures in this context is *relationships between information states*. If human reasoning and inference about the world is to have any point at all, it must be sensitive to the actual content of what is under consideration. Where evidential and inferential relationships are at stake, information content is the first and primary consideration. It is thus no coincidence that a probabilistic framework (which is about content) does a better job of predicting human behavior than the limited structural considerations of classical logic, for example. Of course, it is clear that reasoning will also be influenced by the mechanisms through which it is carried out. However, were these mechanisms to provide greater constraints on, say argument evaluation, than the actual information content of the argument and the relationship of that content to other beliefs, then these mechanisms would necessarily be extremely restricted inferential devices. Our best evidence concerning higher level-cognition suggests that this is not what human thought is like^3^.

For sure, there are deviations from “normative responding” in any reasoning or evidence evaluation context that has been examined, but the deviations would have to outweigh the correspondence to provide greater, more fundamental, and more useful initial constraints. Otherwise, starting from considerations of normative responding will provide the single biggest gain in predictive accuracy. Moreover, via inspection of systematic deviations, it likely provides one of the most powerful routes to identifying where mechanism constraints must be playing a role, and thus to what those mechanisms might be.

## Why normative, why rational?

For many applications of the Bayesian framework the appeal to its normative status is integral. What then does that status rest on, and what kind of rationality or optimality can it consequently bestow?

In fact, there are multiple, independent routes to establishing a normative basis for Bayesian inference (see e.g., Corner and Hahn, 2013 for detailed discussion both of the general issue of normativity and Bayesian inference specifically). Lack of awareness of these distinct possibilities makes it easy to underestimate both the ways in which Bayesian inference may be perceived to provide a norm, that is a prescription of how one *ought* to behave, and to over-estimate how readily alternatives may make a rival claim. At the same time, lack of care in considering exactly what the normative status pertains to runs the risk of overblown normative claims for Bayesian models.

Of the different routes for claiming a normative basis for the Bayesian framework, the Dutch Book argument is the most well-known. A Dutch Book is a combination of bets that can be shown to entail a sure loss. In other words, engaging in a combination of bets that constitute a Dutch Book means necessarily incurring a loss, regardless of how the world turns out. Moreover, this loss is immediate, arising the moment the bet is resolved, not just in the long run (as incorrectly stated in Pothos and Busemeyer, 2013).

The Dutch Book argument provides an instrumental argument for assigning degrees of beliefs in accordance with the probability calculus based on the minimal assumption that incurring a sure loss would be undesirable. Specifically, the argument connects degrees of belief to a (theoretical) willingness to bet by assuming that a person with degree of belief *P* in a proposition *a* would be willing to pay up to £*P* to bet on *a*. The Dutch Book Theorem states that if a set of betting prices violates the probability calculus, then there is a Dutch Book consisting of bets at these prices, that is, a combination of bets that guarantees a sure loss. Being in possession of degrees of belief that violate the probability calculus makes possible Dutch Books and conversely, conformity with the calculus provides immunity from Dutch Books (the so-called converse Dutch book theorem, see e.g., Hajek, 2008).

Bayesian inference (and Bayesian modeling), however, is not just characterized by assignment of probabilities in line with the axioms of probability theory, but also by the use of Bayesian conditionalization for belief revision. That is, Bayes' theorem (which itself follows from the axioms of the probability calculus) is used as an update rule to accommodate new evidence. Analogous, so-called diachronic Dutch book arguments exist for Bayesian conditionalization (see Teller, 1973; and for the converse Dutch book argument, Skyrms, 1993).

To illustrate the nature of Dutch Book arguments with a famous example: Assigning to the conjunction of two events or claims a higher probability (or degree of belief) than is assigned to the less probable of the two—the so-called conjunction fallacy—is, in effect, a logical error. The conjunction of two events, A and B, cannot be true without each of the events being true also, and the event “A and B” cannot occur without the event A and the event B occurring as well. Hence they cannot be *less* probable than the conjunction; failing to realize this makes one Dutch-bookable, as exemplified in Table 1 (see also Newell et al., 2007 for a concrete numerical example). For example, believing it to be more probable that Linda is a bankteller and a feminist, than that she is a feminist (Tversky and Kahneman, 1983) means that a combination of bets could be offered which, if accepted, would imply a sure loss.

**Table 1:** **Dutch book arguments**.

The typical way to present Dutch Books is by presenting propositions, associated betting odds, and outcomes in a table. The left most example in the table below illustrates a bet on *a* for an agent who buys a bet with stake 1$ (i.e., 1$ is the amount won if *a* is true) for the price *q*(a) (*q* as in betting “quotient”); by assumption, the agent's betting quotient is determined by her degree of belief that *a* is true. The table is read in the following way: in the case where *a* turns out to be true, the agent receives 1$ as a payout, but has paid *q*(*a*) for the bet, so her net payoff is *1$ - q(a)*. If *a* turns out to be false, there is no payout, and the agent has simply lost the money she paid for the bet. She will make a profit if *a* turns out to be true and she has paid less than 1$ for the bet (i.e., *q(a)* < 1), and a loss otherwise.
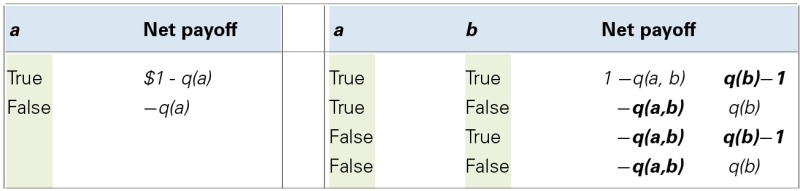
The right hand of the table shows a Dutch Book for the conjunction fallacy. Here, *a* and *b* represent two claims, with *b* representing the less probable of the two. Our agent will *sell* for price *q(b)* a bet that pays out 1$ if *b* turns out to be true, and pay out 0 if it is false. Our agent will also *buy* for price *q(a,b)* a bet that pays out 1$ if the conjunction *(a,b)* is true and 0 otherwise. Because our agent commits the conjunction fallacy *q(a,b)* is greater than *q(b)*. In each row, the net payoff is negative, so whatever the truth or falsity of *a* and *b*, our agent makes a loss. This can be read off directly for rows 2–4 (quantities in bold are “losses,” quantities in plain font are “gains”). In the case of row 1, where both *a* and *b* are true, our agent wins 1$ because the conjunction is true. From this 1$, the price paid for the bet needs to be deducted to calculate net gain. Against this is then set the loss the agent makes by paying out on the win for *b*. This loss necessarily exceeds the gains. (For two positive numbers *x* and *y*, if *x* > *y*, then 1−x < 1−y; also, *y*−1= −(1−*y*); because *q(ab)* > *q(b)* by definition, the gain 1−*q(ab)* must be smaller than the loss *q(b)*−1, meaning a net loss overall).

The example of the conjunction fallacy is chosen here, in part, because it has been argued recently within the cognitive literature that quantum probability may provide a more appropriate framework for modeling human cognition than classical probability (e.g., Pothos and Busemeyer, 2009; Busemeyer et al., 2011). This not only involves the use of quantum probability as a descriptive tool, but its proponents have specifically asked about its normative or rational status (see e.g., Busemeyer and Bruza, 2012; Pothos and Busemeyer, 2013, 2014). For the conjunction fallacy, the ability to model what, from the perspective of classical logic and probability, are viewed as “errors” has been presented as one of the key modeling “successes” within the quantum framework (but see for challenges to its descriptive adequacy e.g., Tentori and Crupi, 2013). However, adherence to quantum probability in this way licenses the conjunction fallacy, and hence, is Dutch-bookable^4^. The Dutch book illustrates why this has traditionally been viewed as a mistake.

Unsurprisingly, in seeking to make their case for “quantum rationality,” Busemeyer and colleague are skeptical about Dutch book arguments and the extent to which they justify a normative status for classical probability. In particular, they highlight a supposed practical limitation of Dutch Book justification: “Avoiding a Dutch book requires expected value maximization, rather than expected utility maximization, that is, the decision maker is constrained to use objective values rather than personal utilities, when choosing between bets. However, decision theorists generally reject the assumption of objective value maximization and instead allow for subjective utility functions (Savage, 1954). This is essential, for example, in order to take into account the observed risk aversion in human decisions (Kahneman and Tversky, 1979). When maximizing subjective expected utility, CP [insertion: CP = Classical Probability] reasoning can fall prey to Dutch book problems (Wakker, 2010)” (Pothos and Busemeyer, 2013, p. 270).

This argument (largely repeated in Pothos and Busemeyer, 2014) conflates two separate issues: whether or not utilities are “subjective” and whether or not an agent is “risk averse.” On the issue of subjective utilities and Dutch books, Pothos and Busemeyer are wrong: The Dutch Book argument could equally be run over subjective utilities (see e.g., Hajek, 2008). In general, the so-called representation theorems for expected utility^5^ are typically defined over preferences- that is subjective valuations (see e.g., Karni, 2014). These representation theorems establish that as long as an agent's preferences respect certain fundamental axioms an expected utility representation of those preferences (which casts them as a combination of probability and utility) is guaranteed. Hence economists long assumed that people's choices might be well-described as “maximizing subjective expected utility.” In their *descriptive* application of expected utility theory, they have also sought to allow for the fact that people are frequently “risk averse”: many might, for example prefer 10$ for sure, over a 50/50 chance of receiving either 30$ or 0$, even though the expected value of the latter option is higher (namely 15$) and picking it will lead to greater gains on average.

Within Expected Utility Theory (EUT) risk aversion can be modeled by assuming that people have non-linear, concave utility functions whereby twice as much money becomes less than twice as “good”^6^. This does not mean that people *should* have non-linear utility functions and be risk averse, however. From the perspective of EUT, risk aversion *costs money*, and the degree to which the concave utility function diverges from a risk neutral, linear, utility function captures an agent's “risk premium,” that is, the price an agent is willing to pay in exchange for certainty over and above expected monetary value. Given that risk aversion implies loss relative to expected value the possibility of Dutch Books under risk aversion seems unremarkable and simply highlights, in a different way, the cost of risk aversion. Risk aversion as a descriptive fact about human preferences does not make a Dutch Book a “good thing”; rather there may be practical contexts in which the price of susceptibility to Dutch Books may be a price an agent is willing to pay in exchange for some greater good. It is thus unclear how risk aversion undermines the Dutch Book argument.

Pothos and Busemeyer's argument is in many ways illustrative of the lively debate about Dutch book arguments. Such debate has focussed to a good extent on how literally one may interpret them and thus how far exactly is their normative reach (for extensive reviews see e.g., Hajek, 2008; for summaries of the main lines of argument see e.g., Corner and Hahn, 2013): for example, one can also avoid a particular Dutch book simply by refusing to bet (though we cannot refuse to bet against nature in general, i.e., we are forced in daily life to make decisions under conditions of uncertainty).

Such arguments do not detract from the fact that the existence of a Dutch book highlights a defect of sorts in a set of probabilities or degrees of belief (e.g., the failure to recognize that if the conjunction is true, each of the conjuncts is necessarily true also). And the defect highlighted (via the theoretical “sure loss”) is one that obtains regardless of the way the world is, that is, what actually turns out to be true or false.

Normative justification for Bayesian probability can thus also be derived from considerations of accuracy (examples of this are Rosenkrantz, 1992; Joyce, 1998; Leitgeb and Pettigrew, 2010a,b). Accuracy-based justifications involve the use of a scoring rule to measure the accuracy of probabilistic forecasts as used, for example, in meteorology, (e.g., Winkler and Murphy, 1968). Scoring rules allow one to assign credit for correct predictions, and penalties for incorrect ones. Overall accuracy is then reflected in the total score. Rosenkrantz (1992) shows that updating by Bayes' rule maximizes the expected score after sampling; in other words, other updating rules will be less efficient in the sense that they will require larger samples, on average, to be as accurate. This holds for any way of measuring accuracy that involves a so-called “proper scoring rule,” that is, a scoring rule which will yield highest scores when agents report “honestly” their actual degrees of belief (that is, there is no incentive for agents to, for example, “hedge their bets” by reporting more conservative estimates than they believe). Furthermore, this optimality of Bayesian conditionalization with respect to maximizing accuracy holds not just for “interest-free inquiry,” but also holds where actions dependent on our beliefs about the world are at stake: using Bayesian conditionalization to update our beliefs upon having sampled evidence maximizes expected utility (Brown, 1976; Rosenkrantz, 1992). Finally, Leitgeb and Pettigrew (2010b) demonstrate that for a common measure of accuracy (the Brier score, Brier, 1950), Bayesianism (i.e., assignment of probabilities in accordance with the probability axioms and updating via Bayes' rule) follows from the simple premise that an agent ought to approximate the truth, and hence seek to minimize inaccuracy. Being Bayesian will minimize inaccuracy of the agent's beliefs across all “possible worlds” the agent is conceptually able to distinguish and hence, in principle, to entertain!^7^

These results provide a normative justification that, unlike the Dutch book argument, is direct: it is the goal of Bayesian inference to make inductive inferences about the world, and such inference is optimal in a well-defined sense, whereby—on average—no other procedure can do better.

What is true of induction in general, of course, can also be applied to specific cases. For example, in the context of supervised categorization, that is, the task of trying to assign instances, including novel instances, to the right (pre-existing) category, the so-called Bayes' optimal classifier will assign items to categories in such a way as to minimize the expected error rate, and thus provides a point of comparison in machine learning contexts (see e.g., Ripley, 1996)^8^.

Considering in such detail various strands of justification for why “being Bayesian” might be viewed as normative or rational is important for a number of reasons. Vis a vis a “normative challenge” such as that by proponents of quantum probability, it makes clear quite how much is required for such a challenge to be well-supported. Merely assuming or speculating that human behavior is rational will never suffice to make it so, and Elqayam and Evans (2011), in particular, have been right to highlight that such an inference from “is” (i.e., how people behave) to “ought” (i.e., how they should behave) would be fallacious [see Elqayam and Evans (2) above]. However, the normative status of Bayesian probability does not rest on its descriptive fit to human behavior, but rather on independent arguments such as those just described.

Furthermore, it is because of these normative foundations, that Bayes' theorem, though conceptually simple, is far from conceptually trivial in the way Jones and Love (2011) might be taken to suggest (3 above). It figures centrally within formal work in the philosophy of science and within epistemology that is concerned with fundamental questions about information seeking, evidence, and explanation, and it figures centrally in statistics, machine learning and artificial intelligence (and that fact, incidentally, adds an interdisciplinary richness to Bayesian models both at the “blue” and the “green” end). For all these disciplines, normative questions about how one ought to behave, or how a problem is best solved, are both theoretically interesting and practically important. Indeed, the debate about Bayesian models itself is a debate about what should count as a “good” theory and about how psychological research “ought” to proceed.

It is thus an interesting question in and of itself how a particular model or procedure relates to an optimal Bayesian one. As a consequence, the theoretical interest and explanatory power of a Bayesian formalization does not rest on whether or not it makes deviant (and hence unique) predictions from existing psychological theories. Contrary to Jones and Love's critique that Bayesian models frequently merely recapitulate extant (mechanism level) theories (2 above) and to Bowers and Davis perception that they rarely “make better predictions” of human behavior than simpler, non-Bayesian models, there may be added value in “mere recapitulation” because it is informative with regard to normative concerns, which in turn opens up the possibility of functional explanations with regard to *why* the system is operating the way it does.

Of course, as outlined earlier in the context of Anderson's rational model, the normative force of Bayesian conditionalization applies only to the extent that Bayesian inference has a clear mapping onto the task under which it is a core component. Where it does, however, viewing a Bayesian formalization and a mechanistic model simply as “competitors” partly misses the point. Furthermore, the normative aspect may give Bayesian formalization a unique role in deriving adequate mechanistic accounts in the first place, as the final section of this paper will seek to show.

## The false tension between mechanism, process models and normative accounts

Running through the critiques of Bayesian modeling that form the focus of the present paper seems to be a perception that “rational” or “normative” considerations are blind to, or even at odds, with mechanism and process-level concerns; however, it may be argued that they are, in fact, part of the route to identifying mechanism or process-level constraints in the first place.

Specifically, it seems likely that pinning down properly cognitive constraints will require appeal to optimality. As Howes et al. (2009) have recently argued, the space of possible cognitive theories is massively under-constrained. The notion of *cognitively bounded rational analysis* provides a means by which to limit that search space in ways that other approaches do not allow, thus providing an essential complement to other methods. Specifically, the study of cognition faces the particular difficulty of humans' inherent flexibility: multiple strategies are typically available for any given task, and the project of seeking to discern cognitive invariants must distinguish between aspects of behavior that appear universal because they, in fact, reflect hard constraints within the system, and those that arise time and again simply because they reflect selection of an obvious, best strategy.

In light of this difficulty, Howes et al. (2009) demonstrate how making strategies computationally explicit, determining their expected pay-offs, and seeking to understand performance relative to those optimal strategies is fundamental to tackling the credit-assignment problem between “fundamental cognitive constraint” and “strategy selection.”

Such an approach seems at odds with critiques of Jones and Love (2011), Elqayam and Evans (2011), and Bowers and Davis (2012a,b). In arguing that process level theories are more important and should be given precedence or that research would advance more quickly without normative theories, these critiques are overlooking the methodological value that stems from the fact that optimal models (in general) form a privileged class of explanation. It is a reasonable default assumption that the cognitive system is trying to do something sensible. Consequently, the fact that a strategy would be optimal supports a presumptive inference to the fact that it is indeed the strategy being used and this has been seen as methodologically important not just in psychology, but also economics and the social sciences.

The standard method of economics has been founded on optimization: Individual agents are presumed to be rational and it is the goal of economic theorizing to understand aggregate behaviors that arise from the interactions of such individuals (see e.g., Lehtinen and Kuorikoski, 2007). Rational choice theory has assumed that economic agents have stable and coherent preferences as set out by expected utility theory (Von Neumann and Morgenstern, 1947). This methodological commitment, though challenged by behavioral economics (see e.g., Thaler and Mullainathan, 2008), has not only been seen as successful within economics, but has been exported to adjacent disciplines such as political science (see e.g., Cox, 1999; Ferejohn, 2002).

Though conceived primarily as a normative theory, expected utility theory has, at times, been viewed as a descriptive theory within economics (see e.g., Friedman and Savage, 1948), and its normative appeal has been viewed as a prima facie reason for why it might provide a descriptive account (Friedman and Savage, 1952 see also Starmer, 2005 for critical discussion). Even now, given overwhelming evidence of violations of rational choice theory in both experiments and field studies (see e.g., Camerer, 1995), the theories of aggregate behavior arising from idealized rational agents aim to be descriptively accurate; this may be possible because certain behavioral contexts provide pressures that lead individuals to utility maximizing behavior (see e.g., Binmore, 1994; Satz and Ferejohn, 1994) and because the behavior of aggregate systems may be robust to the deviations from rational choice theory real agents might display (Lehtinen and Kuorikoski, 2007)^9^. None of this involves a fallacious *ought-to-is* or *is-to-ought* inference of the kind Elqayam and Evans accuse “normativism” of [see Elqayam and Evans (2) above]. Such a fallacy would be commited if one thought the world was a particular way simply because it ought to be, or, conversely, that something out to be the case simply because it was. However, the expectation of rational behavior simply thinks it *likely* that people behave a certain way because they ought to, not that they necessarily do; at the same time, what counts as rational does not rest on whether or not people actually behave the way they should (*is-to-ought*), because the normative claim has been independently derived^10^.

More generally, rational standards provide essential interpretative tools: Any human behavior typically allows many different interpretations, and this is as relevant to science as it is to everyday life. In day-to-day life we resolve ambiguity with “the principle of charity” (e.g., Govier, 1987; see also Oaksford, 2014). Specifically, given multiple interpretations of what someone is saying, we pick the interpretation that renders what they are saying most sensible as our default interpretation. This interpretation may be wrong, and further evidence will force us to abandon it. However, the basic fact that there are default orderings over possible interpretations simplifies massively the task of understanding. Even without specific knowledge of an individual we can typically make reasonably accurate predictions just on the basis of what would be “sensible” (though again, there is no guarantee that these predictions will be correct).

The principle of charity likewise applies to the formal context of understanding behavior within psychological research (see also Hahn, 2011). If we observe something counter-intuitive or surprising, we should as researchers always ask ourselves whether there is an interpretation of participants' behavior that might render it sensible (and hence predictable). Such consideration may identify discrepancies in the way experimenter and participant view the task, leading the researcher to revise interpretations of what it is participants are doing, and many of the seeming “errors” and “biases” have been re-evaluated in this way (see e.g., Hilton, 1995).

This is not an attempt to find rationality at any cost; instead, it is *an interpretative strategy* that provides an essential methodological tool. This is further illustrated by ideal observer analysis as has been hugely successful in the study of perception (e.g., Geisler, 1987). Ideal observer models employ the formal tools of probability and decision theory to specify a model of optimal performance given the available input for a task. Actual human performance is then compared to the performance of this ideal agent. In a process of iterative refinement, human performance and ideal observer are brought into closer and closer correspondence by incorporating capacity limitations of the human system into the ideal observer. This approach provides a tool for the *elucidation* of mechanism and process, embedded in an overall account that seeks to understand the system as “doing the best it can do” given the available hardware. In so doing, the approach inherently links behavioral prediction, mechanistic and functional explanation. In character, it might be viewed as a methodological formalization of the principle of charity.

Crucially, the aim is not to declare the system “optimal” *per se* (see also Griffiths et al., 2012 for related points on Bayesian modeling outside the context of ideal observer analysis). It remains the case that the (truly) optimal agent will be an ideal observer who is not subject to the many constraints of the human, physical system. So, to the extent that the human system achieves less than maximal performance, it is not “optimal” in the strongest possible sense, even if it is doing the best it can. At the same time, in the limit, a model that embodies *all* the constraints of the human system under scrutiny will just *be* that system. This means that, as a theoretical statement, it becomes increasingly vacuous to label a system as “optimal” (even in a weaker sense) as more and more constraints are built into the optimal agent to match its behavior (see also Jarvstad et al., 2014).

Instead, the point of the approach is a methodological one: rational models aide the disambiguation between competing theories and assist in the identification of underlying cognitive universals above and beyond the demand characteristics of experimental tasks (Howes et al., 2009). Once again, this gives such models and considerations a special status, above and beyond degrees of “model-fit” and so on.

## Summary and concluding remarks

It has been argued in this paper that recent critiques of Bayesian modeling, and even more general critiques of computational level theories centered around normative considerations, are misdirected and misjudged. Specific examples have been used to counter any claim that Bayesian modeling would be inherently too flexible and thus unfalsifiable: not just the long-standing literature on judgment and decision-making, but more recent work within the context of reasoning and argumentation (e.g., Harris et al., 2012) provide ready examples of parameter-free model fits, where the model itself consists simply of Bayes' theorem.

It has also been claimed that for the development of “good” cognitive models other constraints (process level, or mechanism level) may be more important; against this, it has been highlighted that in many domains (in particular high-level domains such as reasoning or argumentation) the task participants face is one defined by inferential relationships between information states, and that an account that is based on those informational relationships is thus likely to explain most of the variance in behavioral prediction. That said, Bayesian accounts have been remarkably successful even in areas, such as perception (e.g., Knill and Richards, 1996; Yuille and Kersten, 2006), where mechanism can reasonably be expected to play a key role. Moreover, in many such domains, ideal observer analysis plays a valuable methodological role in identifying and understanding mechanistic constraints (Geisler, 1987). Hence the conflict between “mechanism” or “process” and rational explanation is methodologically ill-conceived. Pinning down processing constraints is likely to actually *require* appeal to optimality (see also, Howes et al., 2009).

At the same time, the present paper has given examples from within the reasoning and argumentation literature whereby Bayesian accounts, focussed on normative considerations, have demonstrably increased the level of behavioral prediction relative to that previously available in the relevant domain of research, and have provided analyses that open up (and first make possible) entirely new empirical programmes (a far cry from the accusation of merely recapitulating extant process/mechanism models). In all of this, this paper has sought to clarify why normative considerations (or considerations of “rationality” or “optimality”) are theoretically interesting and methodologically important over and above behavioral prediction, potentially making a Bayesian model more than just another one of many competitors.

For any, or even all, of the examples used in setting out these arguments, the authors of the original critiques under scrutiny might wish to respond “but those are not the models I had in mind!.” Certainly, Jones and Love (2011) claim only that Bayesian models frequently or maybe even typically exhibit some of the negative traits they perceive. Likewise, Bowers and Davis (2012a,b) supply a wealth of examples in making their case. The point of the present paper, however, is not to argue about whether or not certain perceptions are fair characterizations of the models that the authors of these critiques might have had in mind. Rather the point is to make the case that even if they were, the perceived limitations do not stem from the models *being Bayesian*. There could be a model or even many models for which some or all of the critiques examined here were apt and fair. However, the existence of examples to which the critiques do not apply indicates that it is not the formalism or Bayesian framework *per se* that would be to blame for any such inadequacy. Rather the fault would lie with the framework's particular application.

This matters because it constrains the debate about models. Whether typical or not, the examples chosen in this paper demonstrate that “Bayesian models” is *the wrong level of generality* at which to pitch these critiques. One may dislike specific models (or maybe even the models generally put forward by a specific modeler) and it will always be entirely proper to have debate about what supposedly makes a specific model “bad.” But in order to best advance the quality of the models we as a discipline produce, such debate will need to be considerably more specific than the general critiques of Bayesian modeling examined here.

To some extent, all three of the critiques surveyed may be taken to agree with this, because each has sought to draw distinctions between types of Bayesian modeling [Jones and Love between “Bayesian Fundamentalism and Bayesian Enlightenment,” Bowers and Davis between “Theoretical and Methodological Bayesianism,” and Elqayam and Evans (2013) between “strict and soft Bayesianism”]. However, those distinctions themselves are motivated by the perceptions/claims that have been scrutinized in this paper. To the extent that these claims have been rejected, further classifications (and recommendations depending on them) are rejected also.

Cognitive modeling, however, does need more than debate about specific models. It arguably needs general debate about what exactly makes a model good, and the entire discipline arguably needs a better understanding of what, in general, makes explanation or theories “good” (for critiques of the state of psychological theorizing see e.g., Gigerenzer, 2009). It seems likely that the critiques by Jones and Love (2011), Bowers and Davis (2012a,b), and Elqayam and Evans (2011) evaluated here were motivated in part by disagreement about what aspects are most valuable in a cognitive model or theory. What those aspects should be and what kinds of theories and explanations we should strive for is a pressing issue. It is of great value if the critiques examined have started such debate.

### Conflict of interest statement

The Reviewer, Mike Oaksford, declares that despite being affiliated to the same institution as the author Ulrike Hahn, the review process was handled objectively and no conflict of interest exists. The author declares that the research was conducted in the absence of any commercial or financial relationships that could be construed as a potential conflict of interest.

## Footnotes

^1^Both are pleasing colors in keeping with the fact that the dimension does not reflect value.

^2^This is, of course, not to say that there has been no empirical work on other aspects of the fallacies or on argumentation more generally (for a recent overview see, Hahn and Oaksford, 2012).

^3^Even for a very restricted inferential device, however, there must be constraints on how its outputs “cohere” with those of other components of the systems if the system is to function effectively. This need for coherence once again brings a focus on information content and with it, a role for Bayesian inference (see Griffiths et al., 2012).

^4^In this application of quantum probability to a macro-level entity such as Linda the feminist bankteller. Needless to say, this is not the standard domain of application for the formalism.

^5^These are themselves often used as justifications for a normative basis of probability, see e.g., Armendt (1993).

^6^Though whether this is descriptively adequate seems doubtful, see e.g., Rabin and Thaler (2001).

^7^With the proviso that these possible worlds are finite, a restriction that seems fine for creatures with finite resources and life spans.

^8^Consideration of the optimal Bayes classifier also makes clear that the “rational” force of Anderson's (1991) model increases the more one is willing to view the task of unsupervised categorization as one of discovering underlying, true categories in nature.

^9^Again, the very fact that theories based on assumptions of rationality have come under increasing pressure within economics (both at the individual and the aggregate level, see e.g., Thaler and Mullainathan, 2008; Fox, 2010) is testimony to the fact that optimal models are falsifiable. At the same time, it is important to not confuse the fact that an empirical or theoretical assumption turns out to be wrong, or at some point needs to be replaced in order for a field to progress further, with the claim that greater insight and more rapid development would have been achieved without that assumption (cf. Elqayam and Evans, 2011; Jones and Love, 2011).

^10^That said, one may take issue with Elqayam and Evans construal of the relationship between is and ought in the context of explaining behavior more generally, see Corner and Hahn, 2013, for discussion.
